# Sequential Keratolytic Pre-Treatment Followed by Tirbanibulin in Hyperkeratotic Actinic Keratoses: A Retrospective Comparative Study

**DOI:** 10.3390/ph19060954

**Published:** 2026-06-19

**Authors:** Ilaria Proietti, Vincenzo Coppolelli, Alberto Taliano, Alessandro Colletti, Carmen Cantisani, Giovanni Pellacani, Concetta Potenza

**Affiliations:** 1 Dermatology Unit «Daniele Innocenzi», Alfredo Fiorini Hospital, 04019 Terracina, Italy; proiettilaria@gmail.com (I.P.);; 2 Dermatology Clinic, Department of Medical and Cardiovascular Sciences, University La Sapienza of Rome, 00185 Rome, Italycarmen.cantisani@uiroma1.it (C.C.);; 3 Department of Drug Science and Technology, University of Turin, 10125 Turin, Italy

**Keywords:** actinic keratosis, tirbanibulin, salicylic acid, Decapan, skin

## Abstract

**Background:** Actinic keratosis (AK) is a common premalignant skin disorder associated with chronic ultraviolet exposure and a recognized risk of progression to cutaneous squamous cell carcinoma. Tirbanibulin 1% ointment is an effective short-course field therapy for AK, but its efficacy in hyperkeratotic lesions (Olsen grade II–III) may be limited by reduced drug penetration through a thickened stratum corneum. Keratolytic pretreatment may represent a plausible strategy to improve topical drug delivery in these more challenging lesions. **Methods:** This retrospective chart review included consecutive adults with Olsen grade II–III AK treated in routine clinical practice with either a bland emollient lead-in followed by tirbanibulin (Group A) or salicylic acid 30% ointment pre-treatment (Decapan, Sanitpharma; Milan, Italy) followed by tirbanibulin (Group B). No study-driven procedures or additional visits were implemented. The 14-day bland emollient lead-in used in Group A was part of the routine clinical management applied during the relevant treatment period and was not introduced or retrospectively constructed for the purposes of the present comparative analysis. Outcomes were extracted from de-identified medical records and photographic documentation obtained as part of standard care. For the purposes of analysis, post-treatment evaluations were grouped into predefined windows of 3–6 weeks (T1), 10–14 weeks (T2), and 22–30 weeks (T3), corresponding approximately to 1, 3, and 6 months after treatment initiation. The primary efficacy endpoints were the Actinic Keratosis Area and Severity Index (AKASI) and Total Lesion Count (TLC). Secondary endpoints included quality of life assessed by the Dermatology Life Quality Index (DLQI). **Results:** Both treatment regimens were associated with clinically meaningful improvements in AK severity. At T3, mean AKASI was significantly lower in Group B than in Group A (0.86 ± 0.38 vs. 1.35 ± 0.27; *p* < 0.001), corresponding to reductions from baseline of 60.6% and 36.9%, respectively. Similarly, mean TLC at T3 was significantly lower in Group B than in Group A (4.80 ± 1.5 vs. 6.35 ± 1.6; *p* < 0.001), corresponding to reductions from baseline of 46.7% and 27.0%, respectively. Quality-of-life outcomes also favored the sequential approach, with lower DLQI scores at T3 in Group B compared with Group A (2.9 ± 1.6 vs. 3.8 ± 1.9; *p* = 0.006). Both treatments were generally well tolerated. Although the incidence of local skin reactions (LSRs) was similar between groups, Group B showed lower retrospectively documented composite LSR scores and lower patient-reported discomfort (*p* < 0.001) and lower patient-reported discomfort (*p* < 0.001). **Conclusions:** Sequential keratolytic pretreatment followed by tirbanibulin was associated with greater reductions in disease burden and with lower severity of treatment-related local reactions in this retrospective cohort (Olsen grade II–III). This retrospective study suggests that keratolytic pretreatment may represent a useful adjunctive strategy in hyperkeratotic AK treated with tirbanibulin. Prospective randomized studies are warranted to confirm these findings and to define standardized treatment protocols.

## 1. Introduction

Actinic keratosis (AK) is a prevalent premalignant condition of the epidermis, principally resulting from cumulative ultraviolet (UV) exposure. Its prevalence varies widely depending on geographic latitude, sun-exposure habits, and skin phototype, ranging from approximately 11–26% in Europe and up to 40% in Australia among fair-skinned adults aged over 40 years [1,2,3]. The incidence is higher in males, immunosuppressed individuals (e.g., organ transplant recipients), and those with occupational or recreational outdoor UV exposure [4]. AK most frequently affects sun-exposed areas such as the face, scalp, and dorsal forearms, and is considered a marker of chronic photodamage. Importantly, AK carries a cumulative risk of malignant transformation to cutaneous squamous cell carcinoma (cSCC), estimated at 0.025–16% per lesion per year, with field cancerization further amplifying this risk [5]. Given this malignant potential, timely and effective management of AK is a key priority for skin cancer prevention. Current therapeutic options, ranging from lesion-directed approaches like cryotherapy to field-directed treatments such as topical 5-fluorouracil, imiquimod, diclofenac, and photodynamic therapy, have demonstrated efficacy despite that often at the cost of prolonged treatment durations, marked local skin reactions, and consequently, compromised patient adherence and quality of life [6]. Tirbanibulin 1% ointment has recently emerged as a novel, mechanism-driven topical agent for the treatment of AK [7]. It acts by inhibiting tubulin polymerization and Src kinase signaling, resulting in cell-cycle arrest (G_2_/M phase), p53-mediated apoptosis, and selective cytotoxicity in dysplastic keratinocytes [8]. In two pivotal, identically designed Phase III randomized controlled trials involving 702 patients, a once-daily application for just five consecutive days yielded complete clearance rates of 44% and 54% at day 57, compared to only 5% and 13% in vehicle-treated groups (both *p* < 0.001) [9]. Despite these robust outcomes, recurrence remains a challenge: nearly half of patients who achieved complete clearance experienced lesion recurrence at one year [10]. Nevertheless, the favorable safety profile (mainly mild-to-moderate erythema and scaling) and the brevity of the regimen make tirbanibulin a highly appealing alternative to longer, more irritating treatments [9]. However, hyperkeratotic Olsen grade II–III lesions may represent a specific therapeutic challenge because the thickened stratum corneum is hypothesized to impede topical drug delivery. Nevertheless, direct tirbanibulin-specific evidence comparing drug penetration or tissue distribution in non-hyperkeratotic and hyperkeratotic actinic keratoses is currently lacking. Some evidence suggests that pretreatment techniques, including keratolysis, salicylic acid-based formulations, or curettage, may enhance topical drug delivery and lesion clearance in hyperkeratotic lesions [11].

Despite the mechanistic rationale for reducing hyperkeratosis before field-directed topical therapy, the clinical role of a 30% salicylic acid ointment as a preparatory step before tirbanibulin has not been specifically investigated in patients with Olsen grade II–III AK. In this context, Decapan, a salicylic acid 30% keratolytic ointment, may represent a rational pretreatment strategy aimed at reducing the hyperkeratotic barrier and improving subsequent topical drug delivery. Therefore, the present retrospective comparative study was designed to evaluate whether Decapan^®^ pretreatment followed by tirbanibulin could improve clinical outcomes, tolerability, and patient-reported quality of life compared with a bland emollient lead-in followed by tirbanibulin in patients with hyperkeratotic AK. We hypothesized that salicylic acid-mediated keratolysis before tirbanibulin application may enhance therapeutic response in clinically challenging Olsen grade II–III lesions.

## 2. Results

A total of 137 patients were identified, of whom 120 had complete documentation within the 6-month follow-up window and were included in the primary complete-case analysis: 60 in Group A and 60 in Group B. Seventeen patients lacked complete documentation within the T3 window and were excluded from the primary analysis, including 10 of 70 patients in Group A and 7 of 67 patients in Group B. Baseline demographic and clinical characteristics were well balanced between the two groups (Table 1). Mean age was 75.20 ± 8.07 years in Group A and 73.65 ± 7.49 years in Group B (*p* = 0.278). Male sex accounted for 55.0% and 43.3% of patients, respectively (*p* = 0.201), while Fitzpatrick phototypes I–II were observed in 60.0% of Group A and 46.7% of Group B (*p* = 0.143). Lesions were mainly located on the face (57% in Group A vs. 60% in Group B; *p* = 0.711), with the remainder involving the scalp (43% vs. 40%; *p* = 0.711). Baseline disease severity was also comparable, with no significant between-group differences in AKASI (2.14 ± 0.28 vs. 2.18 ± 0.34; *p* = 0.479) or total lesion count (TLC) (8.7 ± 2.1 vs. 9.0 ± 2.4; *p* = 0.468).

Both treatment strategies were used during substantially overlapping calendar periods between May 2024 and June 2025, although their relative frequency changed over time as prescribing practice evolved. Both treatment strategies were associated with progressive improvement in clinical severity over time, although the magnitude of response was consistently greater in the sequential treatment group (Table 2). Between-group comparisons at T1 and T2 were considered exploratory secondary analyses, whereas the comparison at T3 represented the prespecified primary analysis. In Group A, mean AKASI decreased from 2.14 ± 0.28 at baseline to 1.87 ± 0.37 at T1, 1.58 ± 0.34 at T2, and 1.35 ± 0.27 at T3, corresponding to an overall reduction of 36.9% from baseline. In Group B, AKASI declined from 2.18 ± 0.34 to 1.67 ± 0.35 at T1, 1.21 ± 0.37 at T2, and 0.86 ± 0.38 at T3, corresponding to a 60.6% reduction from baseline. At the prespecified primary timepoint (T3), AKASI was significantly lower in Group B than in Group A (0.86 ± 0.38 vs. 1.35 ± 0.27; *p* < 0.001).

A similar pattern was observed for lesion counts. In Group A, mean TLC declined from 8.7 ± 2.1 at baseline to 7.5 ± 1.9 at T1, 7.1 ± 1.6 at T2, and 6.35 ± 1.6 at T3, corresponding to a 27.0% reduction. In Group B, TLC decreased from 9.0 ± 2.4 to 6.8 ± 1.8 at T1, 5.9 ± 1.3 at T2, and 4.80 ± 1.5 at T3, corresponding to a 46.7% reduction from baseline. At T3, lesion burden remained significantly lower in Group B than in Group A (4.80 ± 1.5 vs. 6.35 ± 1.6; *p* < 0.001). Overall, both AKASI- and lesion-based analyses consistently favored the sequential keratolytic pretreatment plus tirbanibulin strategy.

Patient-reported quality of life findings are reported in Table 3. Baseline DLQI scores were similar between groups (7.1 ± 2.3 in Group A vs. 7.3 ± 2.5 in Group B; *p* = 0.65). At T3, mean DLQI was significantly lower in Group B than in Group A (2.9 ± 1.6 vs. 3.8 ± 1.9; *p* = 0.006), corresponding to an absolute between-group difference of 0.9 points.

Both regimens were generally well tolerated (Table 4). Local skin reactions (LSRs) were common in both groups, occurring in 85% of patients in Group A and 80% in Group B, without a significant difference in incidence (*p* = 0.47). When summarized as the highest composite score recorded during the early post-treatment period, LSR severity was lower in Group B, as shown by a significantly lower mean composite LSR score (4.6 ± 1.8 vs. 5.9 ± 2.0; *p* < 0.001).

Taken together, these findings suggest that sequential keratolytic pretreatment followed by tirbanibulin was associated with greater reductions in AK severity and lesion burden, as well as lower treatment-related discomfort, compared with bland emollient lead-in followed by tirbanibulin.

**Figure 1:**
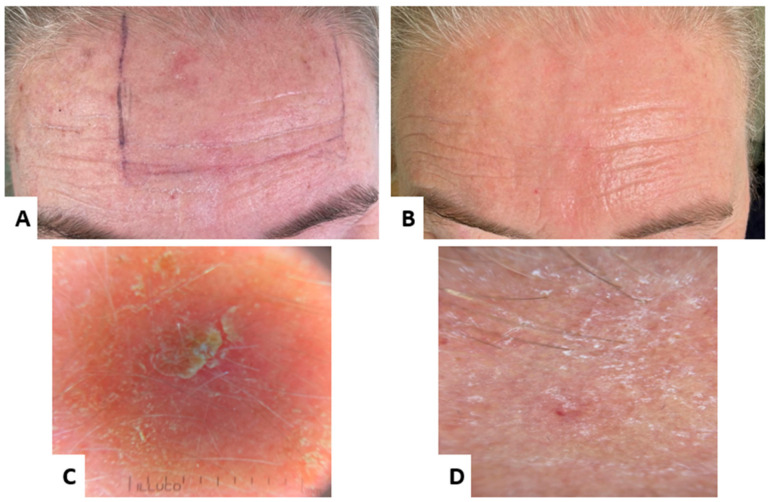
Representative clinical and dermoscopic documentation of hyperkeratotic actinic keratoses treated with sequential keratolytic pre-treatment followed by tirbanibulin. (**A**) Baseline clinical image of a forehead treatment field showing multiple actinic keratoses on chronically photodamaged skin, with erythematous and hyperkeratotic lesions within the marked treatment area. (**B**) Follow-up clinical image of the same anatomical field after keratolytic pre-treatment with 30% salicylic acid ointment followed by tirbanibulin, showing a visible reduction in erythema, scaling, and clinical lesion burden. (**C**) Representative baseline dermoscopic image of a hyperkeratotic actinic keratosis showing adherent yellow-white scale, erythematous background, and superficial desquamation. (**D**) Representative follow-up dermoscopic detail showing attenuation of the hyperkeratotic component, reduced adherent scaling, and residual background actinic damage. All images were obtained as part of routine clinical care, selected for illustrative purposes only, and shown after appropriate de-identification.

Representative de-identified clinical and dermoscopic documentation of the sequential keratolytic pretreatment plus tirbanibulin regimen is shown in Figure 1, illustrating baseline hyperkeratotic actinic keratoses within the treatment field and follow-up attenuation of erythema, scaling, and hyperkeratotic burden.

## 3. Discussion

AK represents one of the most common precancerous dermatologic conditions worldwide, with prevalence rates exceeding 25% in elderly Caucasian populations and a well-established potential to progress to invasive cSCC [12]. Beyond its oncogenic potential, AK significantly impairs patients’ quality of life due to chronicity, frequent recurrences, and the psychosocial burden of visible lesions [13]. For these reasons, effective and tolerable field therapies remain a cornerstone in dermatological practice.

In the present analysis, both regimens demonstrated clinically meaningful efficacy, with the sequential keratolytic plus tirbanibulin regimen achieving significantly lower AKASI scores and lesion counts at 6 months. LSRs, including erythema, scaling, and crusting, were expected given the pharmacodynamic effects of keratolysis and tirbanibulin-induced dysplastic keratinocyte death [14]; However, the lower LSR severity and patient-reported discomfort observed after keratolytic pretreatment appear counterintuitive, because disruption of the stratum corneum might theoretically increase susceptibility to subsequent irritation. Several non-mutually exclusive explanations may be considered. Reduction in the hyperkeratotic and inflammatory surface burden before tirbanibulin may have limited the intensity of the subsequent local response. A thinner and more homogeneous keratin layer may also have promoted a more uniform topical distribution, potentially reducing focal areas of high exposure on an irregular hyperkeratotic surface. In addition, salicylic acid may modulate cyclooxygenase-dependent prostaglandin synthesis and local inflammatory signaling, although persistence of such an effect after the treatment-free interval has not been demonstrated. Finally, residual confounding, differences in lesion biology, assessment timing, and retrospective documentation may have contributed to this finding. These hypotheses were not directly evaluated and require prospective mechanistic investigation. Nevertheless, the lower retrospectively documented LSR scores and patient-reported discomfort observed in Group B are clinically relevant because treatment-related experience may influence adherence, persistence, and overall treatment acceptability [15,16]. An additional consideration concerns the bland non-medicated emollient used during the Group A lead-in. Although it contained no active keratolytic agent, it should not be regarded as completely biologically inert. Hydration of the stratum corneum and softening of surface scaling may have produced modest barrier-modifying effects. Such an effect may have attenuated the contrast between the two treatment strategies, potentially biasing the comparison toward the null, although its magnitude and direction cannot be quantified in this retrospective study.

Our findings are aligned with pivotal trials of tirbanibulin ointment, which have shown effective lesion clearance with an excellent safety profile, characterized predominantly by mild-to-moderate transient LSRs and a negligible rate of treatment discontinuation [9,17]. However, unlike those trials, which primarily assessed short-term lesion clearance in thin (Olsen grade I) AK, our study expands the evidence base by including hyperkeratotic lesions (Olsen grade II–III) and by focusing on patient-reported outcomes and tolerability metrics, which are often underreported in AK clinical research. These aspects resonate with recent calls for more patient-centered endpoints, including DLQI and visual analogue scales for tolerability, to complement traditional efficacy outcomes [1].

Although the between-group difference in DLQI at T3 was statistically significant, its absolute magnitude was modest and below the commonly reported estimates of the DLQI’s minimal clinically important difference. These thresholds are primarily intended to interpret within-patient change and cannot be applied mechanically to differences between group means. Nevertheless, the present result should be regarded as supportive rather than definitive evidence of a clinically perceptible quality-of-life advantage. The DLQI may also have limited sensitivity for detecting small treatment-related differences in patients with actinic keratosis and relatively low post-treatment scores. Future prospective studies should therefore combine the DLQI with an actinic-keratosis-specific instrument, such as the AKQoL, and should report responder analyses based on predefined within-patient improvement thresholds.

**Figure 2:**
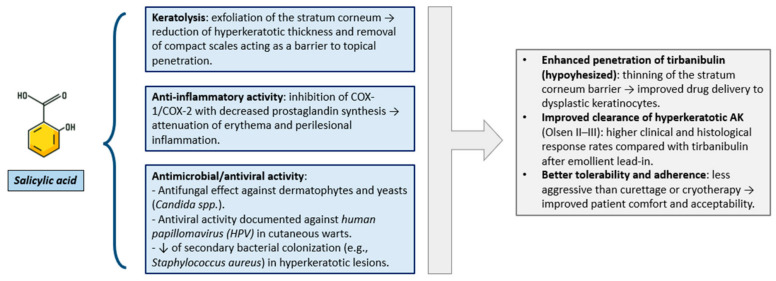
Hypothesized mechanistic framework and observed clinical associations of salicylic acid pretreatment followed by tirbanibulin. The figure distinguishes hypothesized proximal mechanisms from clinical associations observed in the present retrospective cohort. The proposed effects on stratum corneum barrier properties and tirbanibulin distribution were not directly measured.

Beyond clinical efficacy, the mechanistic plausibility of the sequential regimen deserves consideration (Figure 2). Decapan, a 30% salicylic acid ointment, exerts multiple pharmacodynamic effects that may explain the observed differences. Its primary action is keratolysis, promoting exfoliation of the stratum corneum and reducing hyperkeratotic thickness, thereby removing a major barrier to topical penetration [18]. In addition, salicylic acid inhibits cyclooxygenase-1 and -2, lowering prostaglandin synthesis and attenuating erythema and perilesional inflammation [19]. Ancillary activities include antifungal action against dermatophytes and *Candida* spp., antiviral activity against human papillomavirus (HPV) in cutaneous warts, and reduced colonization by bacteria such as *Staphylococcus aureus* in hyperkeratotic lesions [20]. These pharmacological properties provide a biologically plausible rationale for the efficacy findings observed in the Decapan group; however, the mechanisms underlying the lower LSR severity and patient-reported discomfort remain speculative in the absence of a prospective mechanistic assessment.

The 7-day treatment-free interval used in the present cohort represented a pragmatic routine-care choice intended to avoid direct overlap between salicylic acid and tirbanibulin and to allow clinically apparent irritation to resolve before tirbanibulin application. However, the optimal interval between keratolytic pretreatment and tirbanibulin remains undetermined. A shorter interval might preserve a greater degree of keratin-layer reduction but could increase the risk of overlapping irritation, whereas a longer interval might permit greater barrier recovery and potentially attenuate the intended effect on topical delivery. Prospective studies should compare predefined washout intervals and incorporate objective measures of barrier function, such as transepidermal water loss, corneometry, or tape-stripping assessment.

The absence of serious adverse events and the very low discontinuation rate observed in our cohort further reinforce the safety of both regimens. Notably, the improved tolerability profile of the Decapan group may have important clinical implications: in real-world settings, where adherence to prolonged treatment regimens is often suboptimal, regimens with lower tolerability burden are more likely to ensure therapeutic success. This is particularly relevant considering the chronic, relapsing nature of AK and the necessity for repeated treatment cycles over years or even decades.

Strengths of our study include the head-to-head comparative design, the integration of both clinical and patient-reported endpoints, and the focus on tolerability as a determinant of adherence. Nevertheless, several limitations must be acknowledged. First, the relatively short follow-up does not allow for definitive conclusions regarding recurrence rates or long-term control of field cancerization. Second, although statistically significant differences were observed for LSR-related outcomes, larger multicenter cohorts are needed to confirm these findings across diverse patient populations and skin phototypes. The LSR assessments were performed during routine clinical care by the treating dermatologist, who was aware of the prescribed treatment. Although a common six-domain framework was used, the absence of blinded independent assessment may have introduced ascertainment bias. Third, the non-randomized retrospective design may have introduced residual confounding and information bias, particularly for patient-reported outcomes. Given the complete-case approach, the possibility of attrition bias cannot be excluded, especially if missing follow-up documentation was related to treatment response or tolerability. Although Olsen grading was consistently performed by the same dermatologist, possible intra-observer variability inherent to clinical grading cannot be excluded. Finally, although treatment allocation reflected evolving prescribing patterns, both strategies were used during substantially overlapping calendar periods rather than during two discrete treatment epochs. This overlap reduces the likelihood that the observed differences were entirely attributable to separate temporal phases of clinical practice. Nevertheless, secular changes in patient selection, clinician experience, photographic documentation, ancillary skincare recommendations, follow-up intensity, or outcome recording may still have contributed to the findings, and residual temporal confounding cannot be excluded. From a translational perspective, our results underscore the importance of integrating efficacy, safety, and patient-reported outcomes in AK therapeutic decision-making. The field is rapidly evolving, with novel agents such as tirbanibulin providing short-course regimens that minimize LSR burden, while traditional approaches such as 5-fluorouracil, imiquimod, and photodynamic therapy continue to play critical roles in tailored treatment strategies [21]. Future research should aim to refine patient stratification algorithms, incorporating molecular predictors of response, lesion field heterogeneity, and patient preference, in order to deliver fully personalized AK management. Prospective randomized trials are particularly warranted to validate the role of keratolytic pretreatment, confirm its mechanistic benefits, and establish standardized protocols for the integration of Decapan in clinical practice.

## 4. Materials and Methods

### 4.1. Study Design and Participants

This was a retrospective, comparative study conducted at the Complex Operative Unit of Dermatology “Daniele Innocenzi”, Terracina (LT), Italy, aimed at evaluating the effectiveness and safety of sequential keratolytic pretreatment followed by tirbanibulin versus bland emollient lead-in followed by tirbanibulin in patients with Olsen grade II–III actinic keratoses (AK). All visits, assessments, photographs, and treatments were performed as part of routine clinical care; the present analysis was conducted retrospectively on fully de-identified data. No study-driven procedures, interventions, or additional visits were implemented.

**Figure 3:**
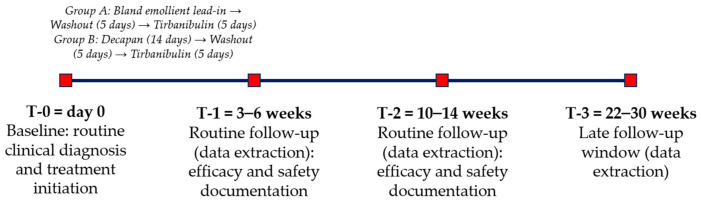
Data extraction framework and predefined post-treatment time windows (T1–T3) used for retrospective analysis.

The study period spanned from May 2024 to June 2025, and eligible patients were identified through a systematic review of institutional dermatology records. Baseline (T0) corresponded to the date of diagnosis and initiation of the prescribed treatment in routine care. Follow-up evaluations were performed according to routine clinical practice and patient needs. For the purposes of this retrospective analysis, post-treatment data were grouped into predefined time windows: 3–6 weeks (T1), 10–14 weeks (T2), and 22–30 weeks (T3) after treatment initiation (Figure 3). When more than one visit occurred within a given window, the evaluation closest to the target time was selected.

**Figure 4:**
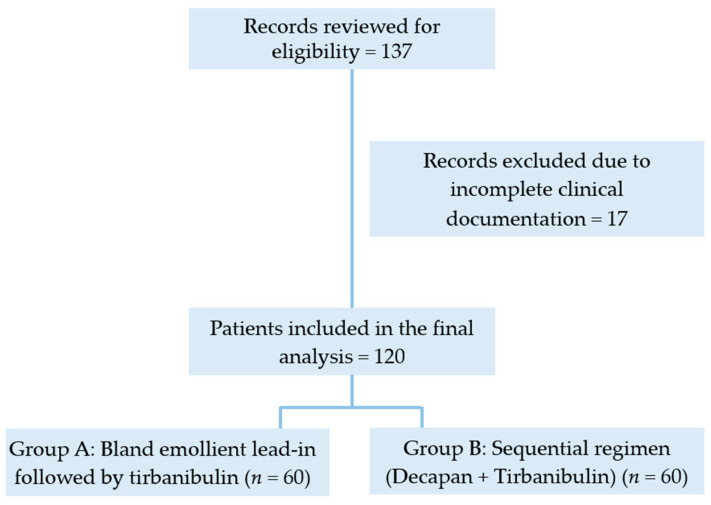
Flowchart of participants in the study.

A total of 137 patients were identified (70 in Group A and 67 in Group B). After exclusion of 17 patients with incomplete follow-up documentation at the 6-month time window (10 in Group A and 7 in Group B), 120 patients were included in the complete-case analysis (60 per group) (Figure 4). Eligibility criteria included: age ≥ 18 years, clinical diagnosis of Olsen grade II–III AK, presence of lesions located on the face and/or scalp, and at least three lesions within a treatment field <25 cm^2^. Exclusion criteria were: (i) concomitant basal cell carcinoma (BCC) or squamous cell carcinoma (SCC), (ii) systemic immunosuppression, (iii) pregnancy or lactation, and (iv) known hypersensitivity to any of the study treatments.

Olsen grading was assigned at baseline by the same board-certified dermatologist through clinical inspection and palpation according to established clinical criteria. Dermoscopy was used to support the diagnosis of actinic keratosis and to identify features requiring additional diagnostic evaluation, but it was not used as a substitute for the clinical assessment of lesion thickness. Because all baseline grades were assigned by a single clinician, an inter-rater reliability assessment was not applicable, although intra-observer variability cannot be excluded. Histological confirmation was not routinely performed for clinically typical actinic keratoses. Lesions considered suspicious for in situ or invasive squamous cell carcinoma were biopsied and excluded from the analysis.

Among the 120 patients included in the final analysis, Fitzpatrick phototypes I–II were observed in 64 patients (53.3%), while phototypes III–IV were observed in 56 patients (46.7%), reflecting a typical Southern European AK population. Most patients reported a long-standing history of chronic sun exposure, either occupational or recreational. With regard to previous treatments, only four patients had never received therapy for AK, whereas the majority had undergone at least one intervention. The most frequently reported modalities included topical piroxicam 0.8% combined with sunscreen (27%), cryotherapy (24%), diclofenac 3% gel (24%), photodynamic therapy (19%), imiquimod 5% cream (15%), 5-fluorouracil 0.5% plus salicylic acid 10% (8%), 5-fluorouracil 5% cream (8%), and 5-aminolevulinic acid patches (4%). Percentages exceed 100% because some patients had received more than one previous AK treatment modality.

Regarding comorbidities, 71 patients (59.2%) presented with at least one chronic condition requiring systemic therapy, while only four (3.3%) had no associated comorbidities and were not receiving systemic medications. The most prevalent conditions were type 2 diabetes mellitus (44.2%), arterial hypertension (41.7%), cardiovascular disease/heart failure (37.5%), dyslipidemia (37.5%), and chronic obstructive pulmonary disease (COPD, 7.5%). Percentages exceed 100% because several patients had multiple comorbidities.

### 4.2. Treatment

Patients received one of two treatment approaches as part of routine clinical care:

- Group A (Bland emollient lead-in followed by tirbanibulin): Bland non-medicated oil-in-water emollient once daily for 14 days (standard non-medicated emollient: an oil-in-water emulsion composed of water, humectants (e.g., glycerin), lipid emollients (oils/paraffins), and emulsifying and stabilizing agents), followed by a 7-day washout, then tirbanibulin 1% ointment once daily for 5 consecutive days on the target area. The emollient contained humectant and lipid components but no active keratolytic agent.
- Group B (Sequential keratolytic + tirbanibulin): Salicylic acid 30% ointment once daily in the evening for 14 consecutive days, followed by a 7-day washout, then tirbanibulin 1% ointment once daily for 5 consecutive days on the target area. The 7-day treatment-free interval reflected routine clinical practice and was intended to avoid direct overlap between salicylic acid and tirbanibulin and to allow resolution of clinically apparent local irritation before tirbanibulin application.

Both treatment regimens consisted of a single treatment cycle. The 6-month follow-up was observational and was intended to assess the durability of clinical response and recurrent or residual lesion burden under routine-care conditions. No additional study-driven treatment cycles were planned within the predefined follow-up windows.

Decapan^®^ (Sanitpharma; Milan, Italy) is a topical keratolytic formulation containing 30% salicylic acid in an ointment base. Its lipophilic unguent vehicle ensures prolonged skin contact and gradual release of the active ingredient, optimizing keratolysis while maintaining adequate tolerability. At high concentrations (20–40%), salicylic acid acts by disrupting corneocyte cohesion, leading to softening and desquamation of hyperkeratotic tissue [18]. In clinical dermatology, ointments of this type are routinely employed for the treatment of hyperkeratotic dermatoses [22].

The rationale for incorporating Decapan lies in the marked hyperkeratosis observed in Olsen grade II–III actinic keratoses, where the thickened stratum corneum acts as a physical barrier that may reduce topical drug penetration. By thinning the keratin layer, Decapan may facilitate deeper penetration of tirbanibulin into dysplastic keratinocytes, potentially enhancing efficacy in more refractory lesions.

Treatment adherence was evaluated retrospectively based on information available in the medical records, including patient-reported use documented during routine visits, as well as notes on returned packaging or residual product. Given the real-world retrospective nature of the study, adherence data were not systematically available for all patients and were analyzed descriptively.

### 4.3. Efficacy Assessment

The primary efficacy endpoints were AKASI and TLC, assessed at baseline (T0) and at follow-up evaluations available within the predefined post-treatment time windows approximating 1 month (T1), 3 months (T2), and 6 months (T3).

The AKASI score is a validated composite tool analogous to PASI in psoriasis, specifically designed to quantify actinic keratoses across the head (face and scalp). It evaluates four anatomical regions (forehead, left cheek, right cheek, scalp), each weighted according to surface area. Within each region, three clinical signs, erythema, thickness, and scaling, are graded on a 0–4 scale, and the proportion of skin involvement is rated from 0 to 6, yielding a total score between 0 and 18 [23]. This provides an objective measure of both disease burden and treatment response.

The TLC represents the absolute number of clinically visible AK lesions in the treatment area, counted by a board-certified dermatologist at each time point. Lesions were identified using dermoscopic confirmation to minimize diagnostic misclassification. TLC captures lesion-level clearance and complements the global severity measure provided by AKASI.

Secondary efficacy endpoints included percentage change from baseline in AKASI and TLC, as well as improvement in health-related quality of life. Quality of life was evaluated using the Dermatology Life Quality Index (DLQI), a validated 10-item questionnaire that assesses symptoms, feelings, daily activities, leisure, work/school, personal relationships, and treatment burden over the previous week [24]. Each item is scored from 0 (not at all) to 3 (very much), with total scores ranging from 0 to 30. Higher scores indicate greater impairment, and cut-offs are generally interpreted as: 0–1 no effect, 2–5 small effect, 6–10 moderate effect, 11–20 very large effect, and 21–30 extremely large effect [24,25].

Standardized digital photographs of the treatment area were obtained as part of routine clinical documentation. For this retrospective analysis, available images were reviewed by two independent dermatologists, blinded to treatment allocation at the time of image evaluation. Representative clinical and dermoscopic images were selected exclusively for illustrative purposes from de-identified routine-care photographic documentation. These images were not used as independent efficacy endpoints; treatment response was assessed using prespecified clinical outcomes, including AKASI and total lesion count.

### 4.4. Assessment of Safety and Tolerability

Safety assessments included both local skin reactions (LSR) specific to field-directed therapy and treatment-emergent adverse events (TEAEs). Safety and tolerability information was extracted from routine follow-up documentation available within the predefined time windows (T1–T3). Additional visits recorded in the charts were included when clinically indicated.

Local skin reaction scores had been recorded during routine clinical visits using a six-domain assessment comprising erythema, scaling/flaking, crusting, swelling/edema, vesiculation/pustulation, and erosion/ulceration. For the present retrospective analysis, the recorded scores were extracted from the clinical charts without post hoc re-grading. Because the assessments were performed in routine care by the treating dermatologist, the assessor was not blinded to treatment allocation, and no inter-rater reliability analysis was applicable [26,27]. Each item was graded on a 4-point ordinal scale (0 = none, 1 = mild, 2 = moderate, 3 = severe), and a composite LSR score was derived by summing individual domain scores (range: 0–18). LSR severity was summarized using the highest composite score recorded during the early post-treatment period. This approach has been validated in phase III studies of tirbanibulin and is considered a reliable method for quantifying treatment-related skin reactions [27].

Treatment-emergent adverse events (TEAEs), defined as any untoward medical occurrence emerging after the initiation of treatment, were extracted from medical records and categorized by severity and relationship to treatment when sufficient information was available. Serious adverse events (SAEs) and adverse events leading to treatment discontinuation were explicitly noted.

Patient-reported tolerability (burning, pain, pruritus) was collected retrospectively from routine clinical documentation. When a quantitative rating (e.g., 0–10 scale) was available in the record, it was used; otherwise, symptoms were summarized descriptively.

### 4.5. Statistical Analysis

Baseline demographic and clinical variables were extracted retrospectively from medical records corresponding to the initial treatment visit (T0). Treatment adherence information, when available, was obtained from documentation recorded during routine follow-up visits. Data were systematically entered into an electronic database and checked for accuracy prior to analysis. Statistical analyses were performed using GraphPad Prism software, version 8.0.2.

Continuous variables were expressed as mean ± standard deviation (SD), while categorical variables were reported as absolute frequencies and percentages. The distribution of continuous variables was assessed for normality using the Shapiro–Wilk test and graphical inspection.

Baseline between-group comparisons were performed using unpaired *t*-tests for approximately normally distributed continuous variables or Mann–Whitney U tests otherwise. Categorical variables were compared using the chi-square test or Fisher’s exact test, as appropriate.

For longitudinal efficacy outcomes, the primary between-group comparison was prespecified at T3 (approximately 6 months) for AKASI and TLC. Between-group comparisons at T1 and T2 were performed as exploratory secondary analyses. For continuous outcomes at each time point, unpaired *t*-tests were used when distributional assumptions were met; otherwise, Mann–Whitney U tests were applied.

Given the retrospective design and incomplete follow-up in a minority of patients, analyses were conducted on a complete-case basis. No formal adjustment for multiplicity was applied to exploratory secondary analyses. In addition, no longitudinal mixed-effects modeling was performed; therefore, post-baseline comparisons should be interpreted with caution, with the prespecified T3 comparison representing the primary inferential analysis.

All statistical tests were two-tailed, and a *p* value < 0.05 was considered statistically significant.

## 5. Conclusions

In this retrospective cohort, sequential keratolytic pretreatment followed by tirbanibulin was associated with greater reductions in AKASI and TLC than the tirbanibulin control regimen in patients with Olsen grade II–III actinic keratoses. The sequential regimen was also associated with lower retrospectively documented LSR severity and lower patient-reported discomfort, although these tolerability findings require cautious interpretation because of the observational design and the limitations inherent to routine-care documentation. This approach can represent a promising strategy for selected patients with hyperkeratotic actinic keratoses; however, its clinical use requires prospective validation and standardization of pretreatment duration, washout interval, application conditions, concomitant skincare, and follow-up. Prospective multicenter randomized trials are needed to confirm efficacy and tolerability and to define an optimized sequential-treatment protocol.

## Figures and Tables

**Table 1:** Baseline demographic and clinical characteristics.

Variable	Group A (*n* = 60)	Group B (*n* = 60)	*p*-Value
Age, years (mean ± SD)	75.20 ± 8.07	73.65 ± 7.49	0.278
Male sex, *n* (%)	33 (55.0%)	26 (43.3%)	0.201
Fitzpatrick type I–II, *n* (%)	36 (60.0%)	28 (46.7%)	0.143
Lesion site: face, *n* (%)	34 (57%)	36 (60%)	0.711
Lesion site: scalp, *n* (%)	26 (43%)	24 (40%)	0.711
Baseline AKASI (mean ± SD)	2.14 ± 0.28	2.18 ± 0.34	0.479
Baseline TLC (mean ± SD)	8.7 ± 2.1	9.0 ± 2.4	0.468

**Table 2:** Change in AKASI and TLC over time and between-group comparisons at each post-baseline time point.

Endpoint	T0	T1	T2	T3	% Change T3 vs. T0	*p* (Between Groups at T1)	*p* (Between Groups at T2)	*p* (Between Groups at T3)
AKASI—Group A	2.14 ± 0.28	1.87 ± 0.37	1.58 ± 0.34	1.35 ± 0.27	−36.9%	*p* = 0.003	*p* < 0.001	*p* < 0.001
AKASI—Group B	2.18 ± 0.34	1.67 ± 0.35	1.21 ± 0.37	0.86 ± 0.38	−60.6%			
TLC—Group A	8.7 ± 2.1	7.5 ± 1.9	7.1 ± 1.6	6.35 ± 1.6	−27.0%	*p* = 0.04	*p* < 0.001	*p* < 0.001
TLC—Group B	9.0 ± 2.4	6.8 ± 1.8	5.9 ± 1.3	4.80 ± 1.5	−46.7%			

The between-group comparison at T3 was prespecified as the primary analysis. Comparisons at T1 and T2 were considered exploratory secondary analyses.

**Table 3:** Dermatology Life Quality Index (DLQI).

Variable	Group A (*n* = 60)	Group B (*n* = 60)	*p*-Value
Baseline DLQI (mean ± SD)	7.1 ± 2.3	7.3 ± 2.5	*p* = 0.65
T3 DLQI (mean ± SD)	3.8 ± 1.9	2.9 ± 1.6	*p* = 0.006

**Table 4:** Safety and tolerability outcomes.

Adverse Event	Group A (*n* = 60)	Group B (*n* = 60)	*p*-Value
LSR, *n* (%)	51 (85%)	48 (80%)	*p* = 0.47
Mean composite LSR score (0–18)	5.9 ± 2.0	4.6 ± 1.8	*p* < 0.001
Discontinuations due to intolerance, *n* (%)	2 (3.3%)	0	*p* = 0.50
Patient-reported discomfort (VAS 0–10)	3.0 ± 1.1	2.1 ± 0.9	*p* < 0.001

## Data Availability

The data presented in this study are not publicly available due to privacy and ethical restrictions related to the retrospective use of clinical records and photographic documentation.
